# Antioxidants, Food Processing and Health

**DOI:** 10.3390/antiox10030433

**Published:** 2021-03-11

**Authors:** Borut Poljsak, Vito Kovač, Irina Milisav

**Affiliations:** 1 Laboratory of Oxidative Stress Research, Faculty of Health Sciences, University of Ljubljana, Zdravstvena pot 5, SI-1000 Ljubljana, Slovenia; borut.poljsak@zf.uni-lj.si (B.P.); vito.kovac@gmail.com (V.K.); 2 Faculty of Medicine, Institute of Pathophysiology, University of Ljubljana, Zaloska 4, SI-1000 Ljubljana, Slovenia

**Keywords:** antioxidants, polyphenols, food processing, health, oxidation, hormesis, stress response

## Abstract

The loss and/or modification of natural antioxidants during various food processing techniques and storage methods, like heat/thermal, UV, pulsed electric field treatment, drying, blanching and irradiation is well described. Antioxidants in their reduced form are modified mainly by oxidation, and less by pyrolysis and hydrolysis. Thus, they are chemically converted from the reduced to an oxidized form. Here we describe the neglected role of the oxidized forms of antioxidants produced during food processing and their effect on health. While natural antioxidants in their reduced forms have many well studied health-promoting characteristics, much less is known about the effects of their oxidized forms and other metabolites, which may have some health benefits as well. The oxidized forms of natural antioxidants affect cell signaling, the regulation of transcription factor activities and other determinants of gene expression. Very low doses may trigger hormesis, resulting in specific health benefits by the activation of damage repair processes and antioxidative defense systems. Functional studies determining the antioxidants’ effects on the organisms are important, especially as reduced or oxidized antioxidants and their metabolites may have additional or synergistic effects.

## 1. Introduction

The oxidation of food, resulting in rancidity, is the second most important cause of food impairment. The first cause is microbial spoilage [1]. The naturally occurring antioxidants in fruits and vegetables scavenge harmful free radicals, and thus have a protective effect against oxidation, improve the nutritional value and prevent food spoilage. Besides, the ingestion of antioxidants prevents intracellular oxidation, which has been associated with health promotion and the prevention of most degenerative diseases [2]. The health-protective properties of fruits, vegetables, culinary herbs and spices also result from the presence of low-molecular antioxidants that protect the cells and their structures against oxidative stress and oxidative damage [3]. Namely, oxidative stress is involved, as a cause or consequence, in over 100 human diseases [4]. A positive correlation between the consumption of whole fruits and vegetables and the prevention of diseases, like atherosclerosis, cancer, diabetes, arthritis, an improved cardiovascular and neurological health, reduced cancer incidence, increased longevity and lowered overall mortality was observed in humans [5,6,7,8,9]. It is unknown if an individual antioxidant or the synergy of several different antioxidants (some of them perhaps undiscovered) in fruits and vegetables contributes to disease prevention. The observed beneficial effects on health might also originate from other phytochemicals present in food, like dietary fiber, folate, vitamins, polyphenols and potassium [10]. The intake of foods that are naturally rich in antioxidants (a daily intake of at least 400 g of fruit and vegetables) has been recommended to the general public by the World Health Organization as a way to protect oneself against chronic diseases [11]; however, there is no formal recommendation for the type, number or amount of antioxidants that should be consumed daily [12].

The enrichment of food with synthetic antioxidants during food processing and preservation is an established method. However, it uses mostly synthetic additives that are not well accepted by modern consumers, who prefer the consumption of natural antioxidants (e.g., tocopherols, carotenoids and ascorbic acid or extracts from rosemary, sage, thyme, savory, marjoram, nutmeg and ginger) and food free of synthetic additives [13].

An alternative to adding synthetic antioxidants (e.g., butylated hydroxyanisole (BHA), butylated hydroxytoluene (BHT), propyl gallate (PG) and tert-butyl hydroquinone (TBHQ)) to foods is to use herbs and spices. Indeed, culinary herbs and spices are a rich source of phytochemicals [14,15] and an antioxidant-dense dietary source [16,17] resulting in a broad spectrum of health promotion activities [17,18]. Herbs, such as dill, garden thyme, rosemary, peppermint, paprika, garlic, curry, chili and black pepper have a high antioxidant activity, some even exceeding the antioxidant activity found in fruits and vegetables [19,20].

Due to their strong antioxidant activity, spices and herbs can inhibit or delay lipid oxidation and thus improve sensorial acceptability and prolong the shelf life of foods [21,22,23,24]. Antioxidants from herbs and spices form complexes with metal ions (Fenton reaction) and/or quench free radicals that are formed during the initiation phase of autoxidation. [21]. A rancid taste and aroma as a result of the lipid oxidation of foods trigger oxidative degradation, leading to a deterioration of food quality and causing undesirable tastes and odors in foods and changes in color and texture [25]. By inhibiting or delaying the onset of lipid oxidation and the development of rancidity in processed foods that undergo various processes during production, natural antioxidants from spices and herbs provide efficient antioxidant protection to minimize the oxidative deterioration of foods [16].

As many studies revealed the decreased availability of health-promoting compounds such as phytochemicals and antioxidants during improper food processing, handling and long-term storage [26], and due to the increased consumer perception, interest and awareness, the food industry currently faces the challenge of whether and which food processing techniques and storage methods should be used to minimize the impact on food quality.

## 2. Processing Techniques That Affect Antioxidant Content and the Oxidative Stability of Food

Many food preservation technologies are used to retain the nutritional attributes of fresh foods and to enhance organoleptic qualities, shelf-life and food safety. Different types of food-processing operations affect the antioxidants and oxidative stability of foods, resulting mostly in losses of antioxidant activity, which occur rapidly during heating or slowly during the storage process [13]. Fruits and vegetables are often subjected to various kinds of processing methods to inactivate microorganisms and to extend shelf life and sensory properties. Such treatment techniques include elevated temperature treatment (pasteurization, sterilization, blanching, evaporation, drying, roasting, frying, microwave heating, infrared heating, ohmic heating), ambient temperature treatment (fermentation, curing, smoking), freezing, high hydrostatic pressure, pulsed electric field, drying/dehydration and food ionizing (gamma and electron beam) and non-ionizing UV-radiation [27]. Each preservation method applied influences the antioxidant content, bioavailability and activity of micronutrients present in fruits and vegetables (Table 1).

For example, thermal processing and long-term storage were shown to reduce the antioxidant activity of fruits [28]. Different cooking methods lowered the antioxidant content of food [29,30]. Long-term frozen storage (12 months) significantly decreased vitamin C and the free radical scavenging capacity, for example in raspberry fruit [31]. Dehydration techniques also affected the phytochemical contents and reduced antioxidant activities, e.g., in berries [32]. Processing methods (soaking and roasting) influenced the total phenolic, flavonoid and antioxidant contents in selected dry beans [33]. High-pressure processing of fruit smoothies significantly affected the antioxidant activity [28] and up to 45% antioxidant capacity was lost after processing fruits into jams [34].

Moreover, the antioxidant activity of black pepper, allspice and oregano was reduced after the treatment at 130 °C for 5 min, with the exception of the increased content of phenolic substances in black pepper [35].

Auto-oxidation and loss of total antioxidant content occur in different fruits and vegetables during food processing and storage. Paradoxically, however, storage or food processing can sometimes improve the antioxidant activity, increase the bio-availability of natural antioxidants, increase their radical scavenging activity [36] and result in the de novo formation of substances with antioxidant properties [37]. For example, some cooking methods can improve the antioxidant capacity of selected vegetables [38,39,40,41]. Cooking (boiling, steaming, microwaving), for example, increased the total phenolic content and antioxidant properties in a pumpkin pulp [42]. The processing techniques, such as microwaving, boiling and roasting, resulted in a 2- to 3-fold increase of the red beet’s antioxidant activity, as compared to the control [43]. Thermal processing increased the total antioxidant activity of tomato and sweet corn [44,45] and elevated the total antioxidant activity and bio-accessible lycopene content in tomatoes, although a loss of vitamin C was observed during the thermal treatment process [46]. Adefegha and Oboh [47] reported that cooking tropical green leafy vegetables decreased the vitamin C contents, while it increased the phenolic content and antioxidant activities. Green mature tomato’s pretreatment by high-voltage electrostatic field (HVEF), a non-chemical technique applied to food preservation with minimal heat production, significantly reduced the contents of superoxide and hydrogen peroxide of tomato during storage and enhanced the activities of antioxidant enzymes including catalase, superoxide dismutase, ascorbate peroxidase and peroxidase. The contents of non-enzyme antioxidant components including reduced glutathione, phenols and ascorbic acid were also increased by HVEF treatment [48]. Additionally, the technique of high-pressure processing, where a pressure from 40 to 1000 MPa is used for 1 to 20 min, resulted in the increased antioxidant activity of carotenoids in tomatoes and orange juices [49] and even increased lutein bioavailability in green beans [50] as well as quercetin and the antioxidant activity in onions [51].

**Table 1:** Food preservation technology can increase, decrease or fail to affect the food’s antioxidant activity.

Food Preservation Technology	Effect on Antioxidants	
	Negative	Positive
Frozen	Reduced vitamin C and free radical scavenging capacity in raspberries frozen for more than 12 months [28].	Retention of vitamin C in raspberry fruit [31].
Dehydration	Reduced antioxidant activity in Saskatoon berries [32].	
Soaking and roasting		Increased antioxidant contents in selected dry beans [33].
High-pressure processing	Reduced antioxidant activity in fruit smoothies [28].	Increased antioxidant activity of carotenoids in tomatoes and orange juices [49]. Increased lutein bioavailability in green beans [50] as well as quercetin and the antioxidant activity in onions [51].
Thermal processing(boiling, steaming, microwaving)	Reduction up to 45% antioxidant capacity after processing fruits (cherry, plums, raspberry) into jams [34]. Loss of vitamin C in tomato [46] and tropical green leafy vegetables [47].	Increased antioxidant activity of red beet [43], tomato [44], sweet corn [45], tropical green leafy vegetables [47].
High-voltage electrostatic field (HVEF)		Enhanced the activity of antioxidant enzymes including catalase, superoxide dismutase, ascorbate peroxidase and peroxidase, and reduced glutathione, phenols and ascorbic acid [48].
γ-irradiation	Ascorbate content of the mango fruits decreased when the dose exceeded 1.5 kGy [52]. Decrease of vitamin C in black pepper, cinnamon, nutmeg, oregano and sage after 10 kGy dose [53].	Increased antioxidant activity in carrot and kale juice after 3 days [54]. No difference in antioxidant activity of turmeric [55]. No difference in antioxidative capacity of cinnamon, ginger, nutmeg, anise, vanilla, licorice, mint [56].
UV-C light		Higher antioxidant capacity in peppers [57], broccoli [58], strawberries [59].
UV-B light		Apple fruit, increased antioxidant capacity in peel, no difference in the flesh [60].
Alkaline pH	Decreased antioxidant activity of aqueous medicinal plant extracts (rosehip: *Rosa canina* L., golden root: *Rhodiola rosea* L., *St John’s-wort: Hypericum perforatum* L. *and the great yellow gentian*: *Gentiana lutea* L.) [61].	

While vitamins C and E are not stable during high-temperature treatments, carotenes [62] and most phenolic compounds become more available in cooked foods [63]. Moreover, the total antioxidant capacity is elevated during the thermal treatment of carotenes and phenolic compounds due to the softening of the food matrix, disruption and degradation of plant cell walls, cellular dehydration and separation of the tissue and release of fibers in fruits and vegetables [63].

## 3. The Neglected Positive Health Effects of Oxidized Forms of Nutritional Antioxidants

Is the antioxidative potential of natural antioxidants the most important biological factor, and does it play a major role in health-beneficial effects? Is the increased amount of oxidative forms of an antioxidant formed during various food processing techniques and storage methods bad on the other hand, from the perspective of human health?

Antioxidants can have dichotomous roles in reactive oxygen species (ROS) production. They are easily oxidized and can act as oxidants to induce damage when present in large concentrations; however, oxidized forms of natural antioxidants are relatively unreactive towards biomolecules. For example, oxidized forms of ascorbate (ascorbate radical and dehydroascorbate) [64,65], phenoxyl radicals [66], tocopheroxyl radicals and lycopene radicals [67] are relatively stable and unreactive and do not cause cellular damage or initiate lipid peroxidation. The damaging free radicals can be generated through Fenton and Haber–Weiss reactions when reduced forms of redox-active metal ions and H2O2 in the presence of antioxidants produce reactive hydroxyl radicals. The limiting factor inside the cells is the H_2_O_2_ that is formed during the autooxidation of synthetic antioxidants like vitamins C, E and others [68,69]. It can reduce free iron Fe^2+^ and other metal ions (chromium, cobalt, copper and vanadium), which initiate free radical generation through Fenton-like reactions.

**Figure 1:**
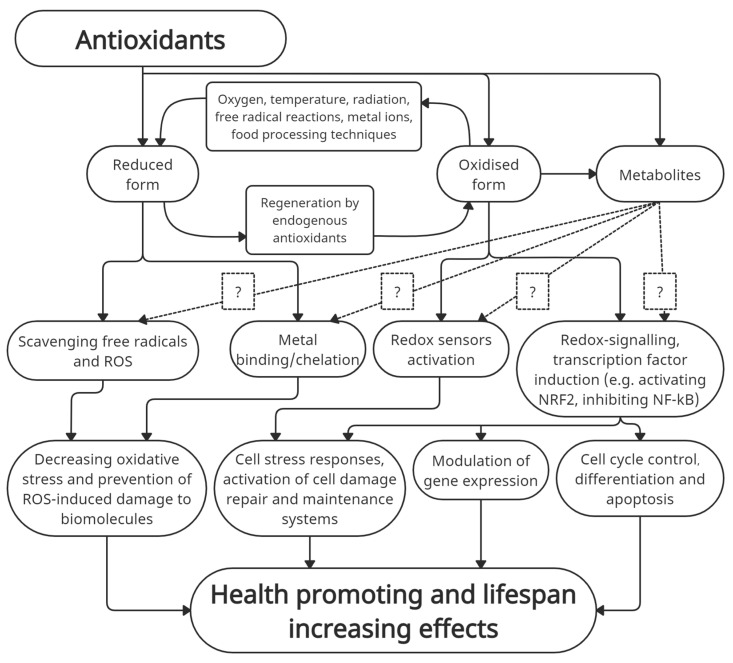
The health-promoting roles of antioxidants and their metabolites.

Antioxidants are involved in immune responses, cell signaling processes, the regulation of transcription factor activities and other determinants of gene expression [70]. Furthermore, ROS interact with cellular signal pathways that control the cell cycle, differentiation and apoptosis [71,72] (Figure 1). Appropriate amounts of oxidized forms of natural antioxidants can modulate cellular metabolism by the induction of cell stress responses and/or the activation of cell damage repair and maintenance systems [73]. Namely, ROS and oxidants are also signaling molecules, e.g., cells use superoxide and hydrogen peroxide as a chemical signal in the regulation of glucose metabolism, cellular growth, proliferation and cell defense against pathogens [74]. Even some lipid peroxidation products modulate signal transduction pathways and induce an adaptive response by upregulating defense processes [75]. An increase in oxidants or ROS can activate the extracellular signal-related protein kinase (ERK), protein kinase B, mitogen-activated protein kinases (MAPKs), insulin receptor kinase and other adaptive stress response pathways like redox-sensitive transcription factors, e.g., nuclear factor-kB (NF-kB) and Activator Protein-1 (AP-1) [76,77,78,79]. Various intracellular redox sensors monitor the redox balance within the cell by detecting the levels of reduced NADH and quinones, the reduced and oxidized glutathione ratio (GSH/GSSG); they can sense the oxidation of reduced species of proteins and low molecular thiols as well as the molecular oxygen levels, reactive nitrogen oxide species (RNOS) amounts [80] and an increase in superoxide, hydrogen peroxide and other ROS. Numerous intracellular signaling pathways are activated by triggering alterations in transcription as a response to increased intracellular oxidation; e.g., direct oxidation and reduction of transcription factors that occur with OxyR or in the APE-1/Ref-1 system, changed subcellular localization of both Nrf2/Keap1 and Yap1 and alterations of intracellular redox buffers that, in turn, modulate the activity of chromatin-modifying enzymes such as SIRT1 or alter the binding of NADH-dependent transcription factors such as BMAL [81]. Moderate concentrations of ROS are essential mediators of defense against pathogens and unwanted cells; the latter is important in cancer prevention [82]. If the administration of antioxidant supplements decreases ROS, it may also interfere with apoptosis and attenuate the elimination of damaged cells, including those that are precancerous and cancerous [83]. Therefore, the excessive neutralization of free radicals by antioxidant supplementation interferes with essential mechanisms of cellular defense and repair [83] and induces so-called “antioxidative stress” (reviewed in Poljsak and Milisav [84]). Oxidized forms of antioxidants at low doses induce the effect of hormesis resulting in increased cellular defense by activating the increased endogenous antioxidant protection and damage repair processes. Hormesis is an adaptive response to a low-intensity stressor exposure that causes an initial disruption in homeostasis [85]. Based on the classical physiological concept of hormesis, Finkel and Holbrook [86] suggested that the best strategy to enhance endogenous antioxidant levels may be oxidative stress itself.

It was observed in some antioxidant-rich beverages, like green tea, black tea and coffee [87,88,89,90], that H_2_O_2_ and superoxide radicals are generated during the auto-oxidation of natural antioxidants under aerobic quasi-physiological conditions [91,92,93]. Ingested low levels of H_2_O_2_ can activate stress response survival pathways [70]. The observed beneficial effects of tea and coffee drinking may be attributed to the ROS-induced mild oxidative stress that triggers cellular adaptive responses [94]. On the contrary, ingesting large doses of exogenous antioxidants can interfere with signaling pathways that regulate cell proliferation, differentiation and apoptosis [95], as well as with the synthesis rate of endogenous antioxidants, like SOD and catalase [96,97,98]; their activity may also be decreased. Large amounts of antioxidant nutrients can increase the oxidative stress in the presence of metal ions due to the Fenton-like chemical reactions [99,100], as antioxidants in their reduced form can induce increased ROS formation or other pro-oxidant effects in the presence of free redox cycling metal ions.

Although the oxidized forms of natural antioxidants may initially deplete endogenous antioxidants (e.g., glutathione), they may also increase the activity of endogenous defense and repair systems as a consequence of the endogenous antioxidants’ regeneration [101]. For example, Hopkins and Morgan [102], Packer and coworkers [103] and May and coworkers [101] demonstrated that ascorbate can regenerate oxidized vitamin E and GSH can regenerate oxidized ascorbate. On the other hand, the ingestion of exogenous antioxidants can interfere with the synthesis of the endogenous antioxidants by suppressing their formation. For example, nutritive antioxidants completely abolished the extension of lifespan by inhibiting an adaptive reaction to ROS called mitohormesis [104,105]. Similarly, reduced health-promoting effects were demonstrated if the subjects exposed to physical activity were treated with antioxidant supplements [106,107]. Supplementation with some antioxidants (e.g., vitamin E and α-lipoic acid) suppresses skeletal muscle mitochondrial biogenesis, regardless of the training status [108]. Antioxidant therapy with vitamins A, C and E and resveratrol can also suppress the synthesis of endogenous antioxidants, thus preventing the beneficial effects obtained with regular exercise [107,109], most probably due to the reduced mitochondrial biogenesis that is stimulated by excessive ROS formation [108]. What is more, supplementation with antioxidants or radical-scavenging supplements could neutralize the ROS that trigger the release of Nrf2 and consequently hormesis during moderate cell stress. Antioxidant supplementation buffers ROS and thus “turns off” the hormesis when the cells’ stress is within the hormesis-inducing range [73].

An important aspect of oxidized forms of natural antioxidants is their cellular uptake. Although few studies investigated this concept, it seems that some antioxidants penetrate cells more sufficiently and faster in their oxidized form. Such an example is vitamin C, through sodium-independent dehydroascorbic acid transport [110]. Dehydroascorbic acid is transported into cells through the glucose transporters Glut1 and Glut2 [111]. The oxidized form of ascorbic acid (dehydroascorbate) is regenerated by intracellular glutathione [112]. Furthermore, an antioxidant silybin, 2,3-dehydrosilybin (DHS), in its oxidized form, enters cells faster, induces adaptive responses and increases tolerance against forthcoming oxidative stress by upregulating endogenous defenses and signaling pathways [113].

## 4. Discussion

The antioxidant activity of fruits and vegetables depends on the type of vegetable/fruit and the processing technology used. The processing approach can decrease, increase or fail to affect the antioxidative content of foods. Emerging technologies, such as high hydrostatic pressure, pulsed electric field, ultrasound, microwave, ohmic heating and irradiation, are being studied as an alternative to the conventional ones in retaining the health benefits of antioxidant compounds in processed food products [44,114]. In general, non-thermal food treatments such as gamma and ultraviolet irradiation, ultraviolet light, pulsed electric fields and high hydrostatic pressure are better in retaining the quality of the bioactive compounds in plant-based foods, while thermal treatments can induce the formation of compounds with new antioxidant properties (e.g., Maillard reaction products) and improve bioavailability (of e.g., carotenes).

We discussed whether the formation of oxidized forms of natural antioxidants during food processing and storage can affect health. Namely, excessive synthetic antioxidant intake (e.g., by ingesting food supplements or adding synthetic antioxidants in food) can alter the endogenous antioxidative defense of cells or cell death pathways as well as suppress the synthesis of endogenous antioxidants [107,109,115]. An increase in cellular antioxidants can trigger increased ROS formation or neutralize free radicals and cause redox dysregulation and signal transduction. A nonselective elimination of free radicals by synthetic antioxidants is more likely to disrupt, rather than extend, the normal cell function. Some compounds with antioxidant activities may improve health, not due to their antioxidant properties, but because of their role in damage repair stimulation and, paradoxically, because of their pro-oxidant activity. The efficiency of defense and repair may be enhanced after the exposure to “moderate” levels of ROS or oxidants, since the expression of many DNA repair enzymes is upregulated during mild oxidative stress or other kinds of stresses [104,116,117].

Low and intermittent doses of oxidants can have a long-term effect on health promotion and life-span increase due to the activation of adaptive response processes and an increase in the synthesis of antioxidant and other defense and repair systems [2]. The beneficial physiological use of ROS is now being demonstrated in different fields, including intracellular signaling and redox regulation. The duration of life-span and health-span may thus be improved by activating the signaling pathways that boost cellular repair and maintenance processes, also with oxidized forms of antioxidants formed during the food processing.

## 5. Conclusions

While a chemical conversion from the reduced to an oxidized form of an antioxidant is an unwanted process, formed during food processing, as regards food quality and nutritional value, such transformation may sometimes result in as yet neglected health benefits. The reduction or increase of phytochemicals does not necessarily result in increased antioxidant activity, and increased antioxidant activity is not always beneficial. Functional studies determining the antioxidant effects on the organisms are important, especially as reduced or oxidized antioxidants and their metabolites may have additional or synergistic effects. As relatively little is known about the cellular effects of oxidized forms of antioxidants, future studies should also evaluate the pro-oxidant activity of vegetables, fruits, culinary herbs and spices and the formation of compounds during food processing and their effect on human health and disease prevention.
